# Direct interaction of the molecular chaperone GRP78/BiP with the Newcastle disease virus hemagglutinin-neuraminidase protein plays a vital role in viral attachment to and infection of culture cells

**DOI:** 10.3389/fimmu.2023.1259237

**Published:** 2023-10-16

**Authors:** Chenxin Han, Ziwei Xie, Yadi Lv, Dingxiang Liu, Ruiai Chen

**Affiliations:** ^1^ College of Veterinary Medicine, South China Agricultural University, Guangzhou, China; ^2^ Zhaoqing Branch Centre of Guangdong Laboratory for Lingnan Modern Agricultural Science and Technology, Zhaoqing, China; ^3^ Integrative Microbiology Research Centre, South China Agricultural University, Guangzhou, China

**Keywords:** Newcastle disease virus, hemagglutinin-neuraminidase protein, GRP78/BiP, attachment, replication

## Abstract

**Introduction:**

Glucose Regulated Proteins/Binding protein (GRP78/Bip), a representative molecular chaperone, effectively influences and actively participates in the replication processes of many viruses. Little is known, however, about the functional involvement of GRP78 in the replication of Newcastle disease virus (NDV) and the underlying mechanisms.

**Methods:**

The method of this study are to establish protein interactomes between host cell proteins and the NDV Hemagglutinin-neuraminidase (HN) protein, and to systematically investigate the regulatory role of the GRP78-HN protein interaction during the NDV replication cycle.

**Results:**

Our study revealed that GRP78 is upregulated during NDV infection, and its direct interaction with HN is mediated by the N-terminal 326 amino acid region. Knockdown of GRP78 by small interfering RNAs (siRNAs) significantly suppressed NDV infection and replication. Conversely, overexpression of GRP78 resulted in a significant increase in NDV replication, demonstrating its role as a positive regulator in the NDV replication cycle. We further showed that the direct interaction between GRP78 and HN protein enhanced the attachment of NDV to cells, and masking of GRP78 expressed on the cell surface with specific polyclonal antibodies (pAbs) inhibited NDV attachment and replication.

**Discussion:**

These findings highlight the essential role of GRP78 in the adsorption stage during the NDV infection cycle, and, importantly, identify the critical domain required for GRP78-HN interaction, providing novel insights into the molecular mechanisms involved in NDV replication and infection.

## 1 Introduction

Newcastle disease (ND), caused by Newcastle disease virus (NDV), is an epidemic disease in poultry farming with significantly economic implications. NDV belongs to the Avulavirus genus within the Paramyxoviridae family and is a single-stranded RNA virus (1, 2). The Paramyxoviridae family also includes other important medical and veterinary pathogens, such as peste des petits ruminants virus (PPRV), measles virus (MV) and canine distemper virus (CDV) (3–6). NDV was first isolated in 1926 from respiratory samples of chickens, initially believed to be the cause of respiratory disease in chickens (7, 8). It causes respiratory and neurological symptoms in poultry, resulting in a high mortality rate.

The mature NDV particles consist of NP (nucleocapsid), P (phospho), M (matrix), F (fusion), HN (hemagglutinin-neuraminidase) and L (polymerase) proteins, as well as two nonstructural proteins (V and W) (9, 10). Certain host nuclear and cytoplasmic proteins, such as nucleolin and microtubule associated proteins, were also identified in the mature NDV particles, and may play a role in the replication and pathogenicity of NDV (11). The HN protein is a multifunctional glycoprotein that exhibits hemagglutinin activity (HA) essential for the aggregation and binding of NDV to cell surface receptors, and neuraminidase activity (NA) that aids in the removal of sialic acid from progeny virus particles and prevents viral self-agglutination (12, 13). As an essential protein involved in NDV invasion of host cells, HN comprises two major structural domains, the N-terminal transmembrane domain and the C-terminal globular head domain, with different regions in these domains playing crucial roles in its function (14–16).

NDV infection triggers multiple cellular stress responses in host cells, including the endoplasmic reticulum (ER) stress, which is associated with viral replication and pathogenesis (17). GRP78/BiP protein, encoded by the HSPA5 gene, belongs to the HSP70 family and serves as a critical molecular chaperone in the ER, playing a vital role in protein folding and quality control (18–20). It consists of two major domains, the nucleotide-binding domain (NBD) in the N-terminal region responsible for facilitating proper protein folding, and the substrate-binding domain (SBD) in the C-terminal region (21, 22). GRP78 can be induced by ER stress, and is present in various cellular locations, including the cell surface, the cytoplasm, mitochondria and the nucleus. As HN can bind to sialic acid receptors on host cell surface to facilitate viral attachment (23, 24), it would be interesting to investigate whether GRP78 may interact with HN and play a functional role in NDV adsorption and entry into host cells.

In this study, we utilized immunoprecipitation and mass spectrometry (IP-MS) analysis to identify potential proteins that interact with HN in NDV-infected DF-1 cells. Among the identified proteins, GRP78 emerged as a key interacting protein. The direct interaction between NDV and GRP78 was subsequently confirmed by validation experiments. Our study further revealed that GRP78-HN interaction may be critical for the attachment of NDV to the host cell surface, an essential step in the NDV replication and infection cycle. These findings suggest that targeting GRP78 would be a promising strategy to combat this viral infection.

## 2 Materials and methods

### 2.1 Cells, virus and antibodies

Human cervical cancer cell line (HeLa), chicken embryo fibroblast (DF-1) and human embryonic kidney cell line (HEK-293T) were cultured in Dulbecco′s modified Eagle medium (DMEM) or Dulbecco′s modified medium nutrient mixture F-12 (DMEM/F-12) containing 10% fetal bovine serum (FBS), 1% penicillin/streptomycin (pen/strep) (Thermo Fisher Scientific, USA), at 37 °C in a humidified atmosphere of 5% CO_2_. NDV virulent strain DHN3 (GenBank: MT447874) was isolated and characterized as previously described (25). Virus titers were determined in DF-1 cells by tissue culture infective dose (TCID_50_), and viral infection was carried out at a multiplicity of infection (MOI) indicated in each experiment.

Rabbit anti-NDV HN polyclonal antibodies (pAbs) were prepared and stored in our laboratory. Rabbit anti-GRP78 pAbs (Proteintech, #11587-1-AP), mouse anti-β-actin monoclonal antibodies (mAbs) (Proteintech, #66009-1-lg), mouse anti-Flag mAbs (Sigma, #F3165), mouse anti-HA mAbs (Abmart, #M20003H), rabbit anti-GAPDH mAbs (Abcam, #ab181602), mouse IgG binding protein conjugated to HRP mAbs (Abmart, #M21001) and rabbit IgG binding protein conjugated to HRP mAbs (Abmart, #M21003) were purchased from the respective companies.

### 2.2 Plasmid construction

For the construction of pXJ40-Flag-C-GRP78, pXJ40-Flag-C-GRP78(1-326aa) and pXJ40-Flag-C-GRP78(327-652aa), the gene fragments of GRP78, GRP78(1-326aa), and GRP78(327-652aa) were amplified from the genomic cDNA of DF-1 cells using PCR. The primers used for amplification are listed in **Table 1**. Subsequently, the fragments were cloned into pXJ40 using the ClonExpress^®^ II One Step Cloning Kit (Vazyme, #C112). To construct pXJ40-HA-C-HN, the NDV HN gene fragment was amplified total RNA extracted from DHN3-infected DF-1 cells by RT-PCR, using primers listed in **Table 1**, and cloned into pXJ40 through homologous recombination. For the construction of pGEX-4T-1-HN, the NDV HN fragment was similarly amplified, using primers GST-HN-F/R (**Table 1**), and cloned into pGEX-4T-1 through homologous recombination. The GST and GST-HN proteins were expressed in *Escherichia coli* BL-21 cells.

**Table 1:** Primers used for construction of plasmids.

Primers	Sequence (5’→3’)	Products
pXJ40-Flag-C-GRP78-F	gactcactatagggcgaattcATGAGGCACCTCCTGTTGGC	2025bp
pXJ40-Flag-C-GRP78-R	cgcctcgagaagcttggatccCTACTTATCGTCGTCATCCTTGTAATCCAACTCATCCTTCTC	
pXJ40-Flag-C-GRP78(1-326aa)-F	gactcactatagggcgaattcATGAGGCACCTCCTGTTGGCGCTG	1047bp
pXJ40-Flag-C-GRP78(1-326aa)-R	cgcctcgagaagcttggatccTTACTTATCGTCGTCATCCTTGTAATCTTCAAATTTGGCACGAGTAAGC	
pXJ40-Flag-C-GRP78(327-652aa)-F	gactcactatagggcgaattcATGGAACTGAATATGGATCTGTTCCGTTCTACGATGAAGCCT	1074bp
pXJ40-Flag-C-GRP78(327-652aa)-R	cgcctcgagaagcttggatccTTACTTATCGTCGTCATCCTTGTAATCCTTATCGTCGTCATC	
pXJ40-HA-C-HN-F	gactcactatagggcgaattcATGGACCGCGTGGTTAACAGAGTCATG	1785bp
pXJ40-HA-C-HN-R	cgcctcgagaagcttggatccTTAAGCGTAATCTGGAACATCGTATGGGTAAACTCTATCATCTTT	
pGEX-4T-1-HN-F	ccgcgtggatccccggaattcATGGACCGCGTGGTTAACAGA	1758bp
pGEX-4T-1-HN-R	gtcacgatgcggccgctcgagTTAAACTCTATCATCTTTGAG	

The sequences with lower-case letter are homologous sequences to the selected vectors and the sequences with upper-case letter are the primer sequences for gene amplification.

### 2.3 Analysis of the interactions between HN and host cell proteins by immunoprecipitation and mass spectrometry

The interactions between target proteins and potential interacting proteins were analyzed using an IP-MS assay. In brief, immunoprecipitation was performed with NDV-infected DF-1 cells (MOI=1) plated in 10 cm cell culture dishes. At 24 hours post-infection (hpi), cells were lysed by adding NP40 lysis buffer, and were subjected to immunoprecipitation using rabbit against NDV HN pAbs and a control antibody against rabbit IgG, respectively, followed by immobilization with protein A/G beads. After adding the antibody-precoated beads to the lysates and incubated overnight at 4°C, the bound proteins were eluted using lysis buffer and subsequently subjected to SDS-PAGE and Western blotting using the corresponding antibodies. Specific protein bands were identified in the silver-stained gel and excised for further analysis. Commercial mass spectrometry (LC-MS/MS) analysis was performed by Beijing Novogene Technology Company. The focus was on analyzing the “high confidence proteins,” the host proteins that exhibit significant interactions (**Table 2**).

**Table 2:** NDV HN-host interacting protein in DF-1 cells (selected proteins).

Uniprot ID	Protein name	Protein function
A0A1D5PXH1	Heterogeneous nuclear ribonucleoprotein A3	RNA-binding and mRNA Splicing-Major Pathway
P0CB50	Peroxiredoxin-1	Cell protection against oxidative stress
E1C0T1	Trafficking from ER to Golgi regulator	Endoplasmic reticulum to Golgi vesicle transport
Q90593	Endoplasmic reticulum chaperone BiP	ER overload response
A0A1X9WEL5	Elongation factor Tu	translation elongation factor activity
Q8JFP1	Eukaryotic initiation factor 4A-II	Protein biosynthesis and RNA binding
O73885	Heat shock cognate 71 kDa protein	Stress response and protein folding chaperone
P17785	Annexin A2	Calcium/phospholipid-binding
A0A1D5PCU1	ADP/ATP translocase	ADP/ATP transmembrane transport
A0A3S5ZPN3	Probable ATP-dependent RNA helicase DDX5	mRNA splicing and nucleotide-binding

### 2.4 Co-immunoprecipitation assay

To validate the interaction between HN and GRP78, HEK-293T cells were co-transfected with pXJ40-HA-C-HN and one of the following plasmids, pXJ40 (vector control), pXJ40-Flag-C-GRP78, pXJ40-Flag-C-GRP78(1-326aa) or pXJ40-Flag-C-GRP78(327-652aa) using Lipofectamine 3000 reagents. At 24 hours post-transfection, cells were lysed, and the lysates were incubated with either EZ view Red Protein A/G (Sigma, #P6486/E3403) or Anti-HA Affinity Gels (Sigma, # E6779) at 4°C overnight, following the manufacturer’s protocol. The agarose beads were washed with NP40 lysis buffer and boiled in a 2× SDS sample buffer (26).

To confirm the interaction between the two proteins in NDV-infected cells, HeLa cells were transfected with pXJ40-Flag-C-GRP78 for 24 hours (h), and were infected with NDV at an MOI of 1 for 24 h. Cell lysates were prepared and treated with or without PNGase F, and immunoprecipitated using anti-Flag Affinity Gels (Sigma, # A2220) to precipitate HN or GRP78 protein from the lysates, and further analyzed by Western blotting with designated antibodies.

### 2.5 Western blotting analysis

Proteins were separated by electrophoresis on 10% SDS-PAGE gels, followed by transfer to a nitrocellulose membrane. The membrane was incubated and probed with appropriate antibodies, and protein bands were visualized using ImageQuant 800 (Cytiva Biosystems, US). The intensities of the target bands were quantified using the Image J program (NIH, USA). As rabbit anti-GRP78 pAbs are not specific to DF-1 cells, most functional and characterization experiments were carried out in HeLa cells either infected with NDV or transfected with plasmid DNA.

### 2.6 GST pulldown assay

To validate the interaction between HN and GRP78, GST and GST-HN recombinant proteins were expressed in *Escherichia coli* BL-21 cells and purified using the GST Fusion Protein Purification Kit (Genscript, #L00207) as per the manufacturer’s protocol. The Glutathione Sepharose 4B beads (Cytiva, #17075601) were incubated with the purified GST-tagged proteins at 4°C for 8 h, followed by washing with lysis buffer for four times, and further incubated overnight at 4°C with GRP78 protein cell lysates. The interaction complexes were eluted from the beads. The presence of GST, GST-HN and GRP78 was confirmed by Western blotting using mouse anti-GST mAbs and rabbit anti-GRP78 pAbs, respectively.

### 2.7 Confocal microscopy assays

HEK-293T cells seeded on a confocal dish were allowed to grow for 24 h to form monolayers, and were transfected with pXJ40-Flag-C-GRP78 or pXJ40-HA-C-HN using Lipofectamine 3000 reagents, following the manufacturer’s instructions. At 24 h post-transfection, cells were fixed with 4% paraformaldehyde at room temperature for 10 minutes, permeabilized with 0.5% Triton X-100 for 10 minutes and then blocked with 5% BSA for 1 h. For immunostaining, the cells were incubated with mouse anti-Flag mAb and rabbit anti-HN pAb at a dilution of 1:500 for 1 h, and then stained with a secondary antibody conjugated with Alexa Fluor 488 or Alexa Fluor 594 at a dilution of 1:1000. Nuclei were counterstained with 4,6-diamidino-2-phenylindole (DAPI) obtained from Thermo Fisher Scientific. The stained cells were visualized using a confocal laser scanning microscope, Stellaris 5 fluorescence microscopy (Leica Microsystems, Germany). All images were captured and processed using Leica Application Suite X (Leica Microsystems).

### 2.8 RNA extraction and quantitative real-time PCR

Total RNA was extracted from samples using the Trizol reagent (Invitrogen, #15596026) following the manufacturer’s instructions, and the RNA concentration was measured using a NanoDrop 2000 spectrophotometer (Thermo Fisher Scientific, USA). Subsequently, cDNA was synthesized from the total RNA using the PrimeScriptTM RT reagent Kit with gDNA Eraser (TaKaRa, #RR047A) according to the manufacturer’s protocol.

Viral RNA copy numbers were determined by RT-qPCR analysis using the HiScript II One Step RT-qPCR SYBR Green Kit (Vazyme, China). qPCR analysis of NDV, GRP78 and GAPDH mRNA were performed with the TB Green^®^ Premix Ex TaqTM II (Takara, #RR820A) and analyzed using the Applied Biosystems™ QuantStudio™3 (Applied Biosystems, US). The following primer pairs were used for mRNA detection: NDV mRNA: Forward primer: 5′-GGAAGGAAGCGGAGCCATCATG-3′, Reverse primer: 5′-GCTGTGGAGGGTTCATCTCATTCG-3′; human-GAPDH mRNA: Forward primer: 5′- TGACATCAAGAAGGTGGTGAAGCAG-3′, Reverse primer: 5′- GTGTCGCTGTTGAAGTCAGAGGAG-3′; human-GRP78 mRNA: Forward primer: 5′- CCAAGAACCAGCTCACCTCCAAC-3′, Reverse primer: 5′- TGAACGGCAAGAACTTGATGTCCTG-3′. chicken-GAPDH mRNA: Forward primer: 5′- CAGAACATCATCCCAGCGTCCAC-3′, Reverse primer: 5′- CGGCAGGTCAGGTCAACAACAG-3′; chicken-GRP78 mRNA: Forward primer: 5′- GCTGTTCAGGCTGGTGTTCTCTC-3′, Reverse primer: 5′- TCCAACTGTCTCAATGCCAAGTGTC-3′. The mRNA levels of the specific genes were determined by normalizing to the internal reference GAPDH and comparing to the mock-treated controls. The fold change was calculated using the 2^^(-ΔΔCt)^ method. The experiments were performed in triplicate, both technically and biologically (27).

### 2.9 RNA interference and Cell viability assay

HeLa and DF-1 cells were plated in 6-well plates and cultured in DMEM supplemented with 10% FBS for 24 h. When the cell confluence reached approximately 80%, HeLa cells were transfected with specific siRNAs using Lipofectamine RNAi MAX according to the manufacturer’s instructions (28). The sequences of siRNAs used in this study are listed as follows: siRNA human-GRP78 1#: GGAGCGCAUUGAUACUAGA; siRNA human-GRP78 2#: GAGGUAAACUUUCCUCUGA; siRNA human-GRP78 3#: GGGCAAAGAUGUCAGGAAA. The sequences of siRNAs used in DF-1 cells are listed as follows: siRNA chicken-GRP78 1#: CCUGACAAAGAUGAAAGAA; siRNA chicken-GRP78 2#: CCAAGGACAAUCAUCUUCU; siRNA chicken-GRP78 3#: GAAGACAAAGAAACAAUAG. These siRNAs targeting GRP78 and siRNA negative control (siRNA-NC) were designed and synthesized by Ribobio (Guangzhou, China). To investigate the knockdown efficiency, the GRP78 mRNA level was assessed by RT-qPCR, and the protein level in HeLa cells were determined by Western blotting, utilizing a rabbit anti-GRP78 pAbs.

Cell viability was assessed using a cell counting kit-8 (CCK-8) (Sigma, #96992) following the manufacturer’s instructions (29). Briefly, HeLa and DF-1 cells were seeded on a 96-well plate at a density of 5000 cells per well, transfected with siRNAs and incubated at 37°C for 36 h, and 10 μL of CCK-8 solution was added to each well. After incubation for 2 h, the optical density at 450 nm (OD_450_) was measured using a microplate reader.

### 2.10 Viral adsorption and entry assay

HeLa and DF-1 cells were transfected with pXJ40-Flag-C-GRP78 (Flag-GRP78) for 24 h or siRNA GRP78 for 36 h, and incubated with NDV (MOI=1) at 4°C for 1 h to allow for virus adsorption. Following by washing three times with chilled PBS to remove any unbound virus, total RNA was extracted using the Trizol reagent, and the levels of NDV mRNA and GRP78 mRNA were quantified by RT-qPCR.

For the NDV entry assays, HeLa and DF-1 cells were transfected with pXJ40-Flag-C-GRP78 (Flag-GRP78) for 24 h or siRNA GRP78 for 36h. After transfection, cells were infected with NDV (MOI=1) at 4°C for 1 h, followed by shifting to 37°C for another 1 h after thorough washing to facilitate viral entry. Cells were then washed with chilled PBS to remove any unbound virus, and harvested for quantification of NDV mRNA and GRP78 mRNA using RT-qPCR.

### 2.11 GRP78 antibody inhibition assay

HeLa cell monolayers in 6-well plates were incubated with either 1μg or 3μg of rabbit anti-GRP78 pAb, or rabbit IgG antibody (as a control), at 37°C for 1 h. Cells were washed three times with chilled PBS, and incubated with NDV (MOI=1) at 4°C for 1 h. Following three washes, the cells were cultured in DMEM at 37°C for 24 h. The levels of NDV mRNA were quantified by RT-qPCR to assess the effects of rabbit anti-GRP78 pAbs on NDV infection of host cells. Additionally, the culture supernatants were collected for TCID_50_ assay.

### 2.12 Statistical analysis

Data were presented as the mean ± standard deviation (SD) of at least three independent experiments. Statistical analyses were performed using GraphPad Prism software. Student’s t-test was used to compare two independent groups when the data followed a relatively symmetric distribution without significant departures from normality. One-way ANOVA models were used to compare three or more independent groups. A p-value of 0.05 or less was considered statistically significant.

## 3 Results

### 3.1 Immunoprecipitation and mass spectrometry analysis of the interactions between HN and host proteins in NDV-infected cells

**Figure 1:**
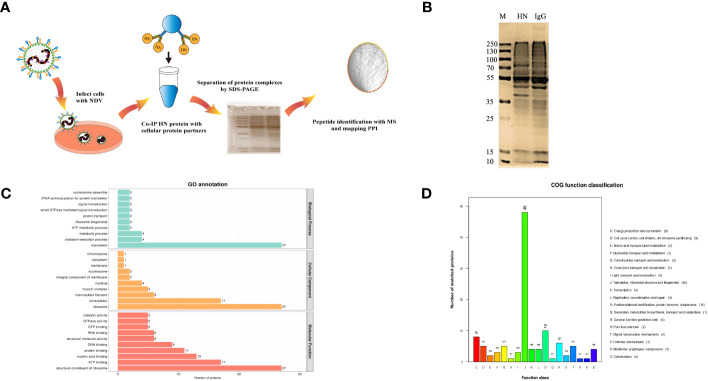
Identification and analysis of host cell interacting proteins with NDV HN proteins. **(A)** Schematic diagram showing the IP-MS procedure for mapping HN-host protein interactomes. **(B)** Separation and silver staining of HN-interacting proteins from NDV-infected DF-1 cells. **(C)** GO annotation of HN-interacting proteins. GO annotation was used to classify HN-interacting proteins in DF-1 cells. The horizontal coordinate represents the number of proteins and the vertical coordinate represents the GO annotation. **(D)** COG annotation of HN-interacting proteins. COG function classification was used to classify HN-interacting proteins in DF-1 cells. The horizontal coordinate represents the functional classification of the annotation and the vertical coordinate represents the number of proteins annotated to the corresponding function.

To identify host proteins that may interact with HN protein, we employed IP-MS analysis of HN protein complexes (**Figure 1A**). In DF-1 cells infected with NDV (MOI=1) for 24 h, HN protein complexes were immunoprecipitated using rabbit anti-HN pAbs and rabbit IgG control antibodies, and checked by silver-staining after being separated on SDS-PAGE (**Figure 1B**). The precipitated proteins were subsequently subjected to MS analysis to identify the interacting proteins.

All the identified proteins were categorized into three main types of annotations: biological processes, cellular components and molecular functions, which were obtained from the Gene Ontology (GO) consortium website. In terms of biological processes, translation emerged as the top one associated with HN (**Figure 1C**), indicating its potential role as a key regulator in NDV infection. The differentially expressed proteins corresponding to significantly enriched molecular functions were predominantly involved in various molecular binding and constituent activities, such as nucleic acid binding, ATP binding, and structural constituents of the ribosome (**Figure 1C**). These findings suggest the involvement of these proteins in viral translation. To further annotate the protein functions, the major identified proteins were classified into 18 categories based on the Cluster of Orthologous Groups (COG) functional annotation. Notably, the categories of translation, ribosomal structure and biogenesis stood out prominently (**Figure 1D**), highlighting the importance of these proteins in these specific functional categories.

**Figure 2:**
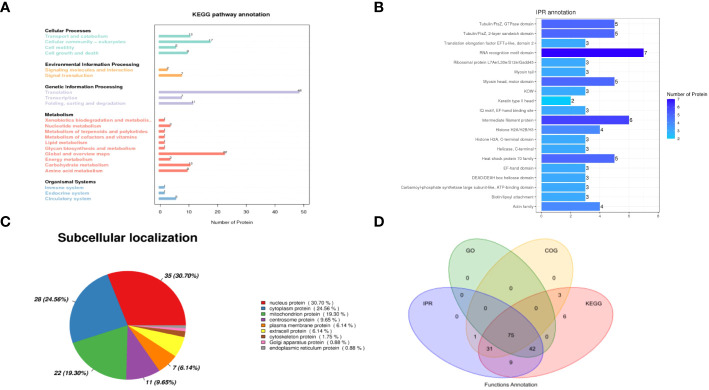
Analysis of interacting proteins with NDV HN proteins. **(A)** KEGG functional annotation. The horizontal axis represents the number of proteins and the vertical axis represents the KEGG pathway annotation. HN-interacting proteins in DF-1 cells were classified based on the KEGG pathway annotation. **(B)** Analysis of the interacting proteins with NDV HN proteins using IPR functional annotation. The horizontal axis represents the number of proteins and the vertical axis represents the IPR annotation. HN-interacting proteins in DF-1 cells were classified based on the IPR annotation. **(C)** Subcellular localization analysis. HN-interacting proteins in DF-1 cells were classified based on their subcellular localization. **(D)** Venn diagram illustrating the collectively enriched proteins. Each circle in the graph represents the annotation results for a database. The overlapping and the non-overlapping sections represent proteins annotated to multiple databases together and individual databases, respectively.

To investigate the signaling pathways in which the identified proteins may participate, KEGG pathway analyses were conducted. Among the various categories, the top three pathways with the highest protein representation were primarily related to translation, global overview maps, and cellular community-eukaryotes (**Figure 2A**). It suggests that these signaling pathways were predominantly activated and may be involved in the host-NDV interaction. Furthermore, InterPro (IPR) annotation revealed the significant expression of proteins associated with the RNA recognition motif domain, intermediate filament protein, Tubulin/FtsZ domain and heat shock protein 70 family (**Figure 2B**). Regarding their subcellular localization, the identified host cell proteins were primarily located in the nucleus (30.70%), cytoplasm (24.56%), mitochondrion (19.30%), and centrosome (9.65%) (**Figure 2C**). By utilizing four analytical methods (GO, COG, KEGG, and IPR), we applied bioinformatics approaches to identify valuable proteins. Through a Venn diagram analysis, a total of 75 collectively enriched proteins were revealed (**Figure 2D**). This comprehensive comparison of the NDV HN-host protein interactome would be crucial for understanding the general mechanisms underlying the regulation of NDV infection and discovery of potential host targets.

### 3.2 Mapping the NDV HN-host protein interactome

**Figure 3:**
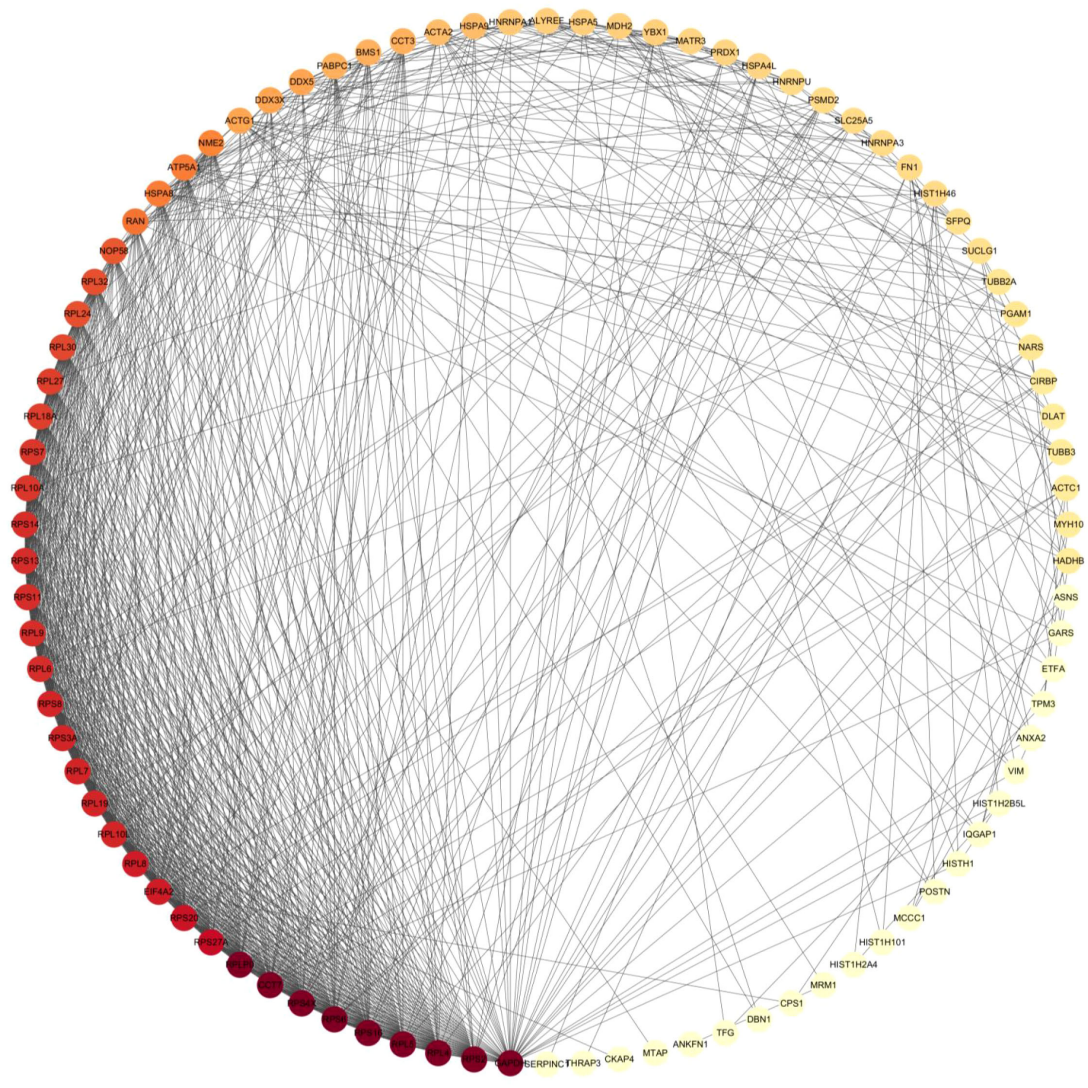
The interactome map of HN-host protein interactions was generated using Cytoscape software. The map displays the interactions between NDV HN-associated host proteins. Red and yellow nodes represent host proteins with high interaction abundance and intermediate abundance, respectively. The incorporation of host factor interactions was based on interaction data obtained from STRING.

To gain a more comprehensive understanding of the HN interactome, we employed the STRING database to analyze the interactions among the HN-interacting host proteins. We successfully identified 167 protein-protein interactions involving HN protein in chicken DF-1 cells. The HN-host interactome was visualized based on MS data and STRING analysis, as depicted in **Figure 3**. Based on the functional categorization of the identified proteins, we have compiled a list of proteins likely to be involved in the replication of the virus, which is presented in **Table 2**. Notably, ribosome-related proteins, heat shock proteins and histone proteins emerged as three major categories in the HN-host interactome. In the subsequent sections, the HN-GRP78 interaction and its potential relevance to NDV infection and replication were systematically studied.

### 3.3 Verification of the interaction between GRP78 and NDV HN protein, and mapping the interacting domain(s) in GRP78

**Figure 4:**
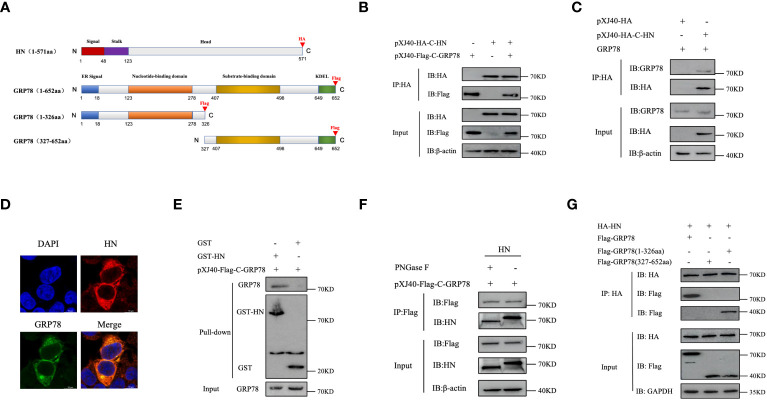
Identification and characterization of GRP78-HN interaction. **(A)** Schematic diagram illustrating the domain structures of HN and GRP78 as well as truncated GRP78 constructs. The functional domains in HN and GRP78 are shown with different colored boxes, and the positions of the HA and Flag tags added to the two proteins are indicated. **(B)** Interaction between HN and GRP78 in HEK-293T cells overexpressing the two proteins. Cells transfected with pXJ40-HA-C-HN and pXJ40-Flag-C-GRP78 were immunoprecipitated using anti-HA agarose beads, and the precipitated proteins were subjected to Western blotting with anti-HA and anti-Flag mAbs. β-actin was used as a control. **(C)** Interaction between HA-HN and the endogenous GRP78. HEK-293T cells transfected with pXJ40-HA-C-HN and pXJ40 vector were immunoprecipitated using anti-HA agarose beads, and the precipitated proteins were subjected to Western blotting with anti-GRP78 pAbs and anti-HA mAbs. β-actin was used as a control. **(D)** Co-localization of GRP78 and HN proteins in HEK-293T cells. Cells overexpressing Flag-GRP78 (green) and HA-HN (red) were immunostained with mouse anti-Flag monoclonal antibody and rabbit anti-HN pAb, respectively, and examined by confocal microscopy. Nuclei were stained with DAPI (blue). **(E)** Analysis of the direct binding of recombinant HN protein to GRP78 protein by GST-pulldown assay. The recombinant proteins were purified using GST beads and detected by Western blotting. The purified recombinant proteins were coupled to GST beads, with GST alone as a control. **(F)** Effects of the N-linked glycosylation of HN protein on its interaction with GRP78. Total cell lysates expressing HN were treated with or without PNGase F, immunoprecipitated using anti-Flag agarose beads, and the precipitated proteins were analyzed by Western blotting with anti-Flag mAb and anti-GRP78 pAb. β-actin expression was used as an internal loading control. **(G)** Interaction of the GRP78(1-326aa) domain with HN protein. HEK-293T cells co-transfected with HA-HN and Flag-GRP78, Flag-GRP78(1-326aa), or Flag-GRP78(327-652aa) were immunoprecipitated and analyzed by Western blotting using anti-Flag mAb and anti-HA mAb. GAPDH expression was used as an internal loading control.

In order to validate the interaction of HN with GRP78 and to investigate the specific GRP78 domain(s) responsible for its interaction with HN protein, we generated the C-terminally HA-tagged HN, C-terminally Flag-tagged GRP78, and a series of GRP78 deletion mutants, containing domains in each part of the GRP78 protein (**Figure 4A**). The C-terminally HA-tagged HN and C-terminally Flag-tagged GRP78 cloned into pXJ40 expression vector were transfected into HEK-293T cells and analyzed by Co-IP assay. It revealed that the exogenous GRP78 protein indeed interacted with HN, as evidenced by the detection of the respective proteins in cells co-expressing the two proteins using anti-Flag and anti-HA monoclonal antibodies (**Figure 4B**). To corroborate the interaction between the endogenous GRP78 and HN, IP assay was further performed in cells overexpressing HA-HN, confirming the interaction between the overexpressed HA-HN and the endogenous GRP78 (**Figure 4C**). Moreover, co-localization between GRP78 and HN was observed in HEK-293T cells by confocal microscopy (**Figure 4D**).

To analyze if glycosylation of HN is required for the interaction between the two proteins, GST pull-down assay utilizing purified GST and GST-HN proteins was conducted, clearly demonstrating a direct interaction between GST-HN and GRP78, as observed in the pull-down assay (**Figure 4E**). Furthermore, total cell lysates expressing HN proteins were treated with or without PNGase F, HN or GRP78 protein was then immunoprecipitated from the lysates using anti-Flag agarose beads. The results of the IP assay confirm that the de-glycosylated HN protein indeed interacted with GRP78 (**Figure 4F**).

To identify the GRP78 domain involved in the interaction with HN, we co-transfected plasmids encoding Flag-C-GRP78, Flag-C-GRP78(1-326aa), or Flag-C-GRP78(327-652aa) along with HA-C-HN, followed by a Co-IP assay. The results demonstrated that GRP78(1-326aa) was capable of interacting with HN protein, while GRP78(327-652aa) failed to exhibit such an interaction (**Figure 4G**). In conclusion, these findings provide robust evidence of the direct interaction between GRP78 and HN.

### 3.4 NDV infection upregulates GRP78 protein and mRNA levels

**Figure 5:**
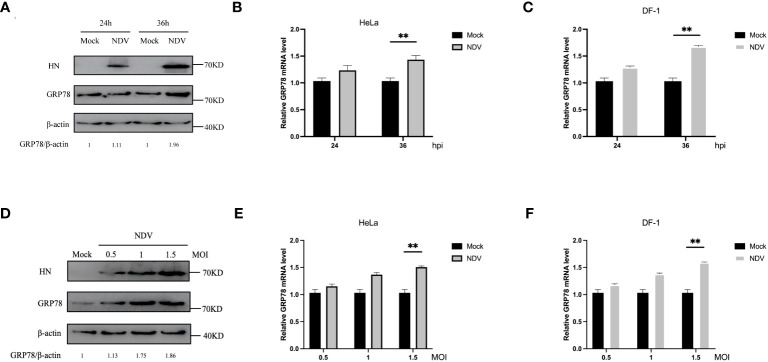
Induction of GRP78 protein expression in NDV infected-HeLa cells and DF-1 cells. **(A)** HeLa Cells were infected with NDV at an MOI of 1, and the GRP78 and HN protein levels were assessed by Western blotting with GRP78 and HN antibodies at 24 hpi and 36 hpi. β-actin expression served as the internal loading control. **(B, C)** Relative expression levels of GRP78 mRNA were determined by RT-qPCR at 24 hpi and 36 hpi, respectively, in HeLa **(B)** and DF-1 cells **(C)**. **(D)** HeLa cells were infected with NDV at an MOI of 0.5, 1 and 1.5, respectively, and the GRP78 and HN protein levels were assessed by Western blotting at 24 hpi. **(E, F)** GRP78 mRNA levels in HeLa **(E)** and DF-1 **(F)** cells infected with NDV at an MOI of 0.5, 1 and 1.5, respectively, were determined by RT-qPCR.All results are presented as the mean ± SD of data from three independent experiments. **p < 0.01.

To study the functional impact of this interaction on NDV infection and replication, we first examined if the expression of GRP78 would be regulated in the response to NDV infection. HeLa and DF-1 cells were either mock-infected or infected with NDV at an MOI of 1, harvested at 24 and 36 hpi, respectively, and subjected to Western blotting to determine the protein level of GRP78 (**Figure 5A**), with β-actin protein serving as a loading control. In parallel, RT-qPCR was performed to assess the mRNA level of GRP78 (**Figures 5B, C**). Our data demonstrated that the protein level of GRP78 was upregulated at 36 hpi in the response to NDV infection. Similarly, HeLa cells and DF-1 cells were infected with NDV at an MOI of 0.5, 1, or 1.5, and the assays were performed at 24 hpi. Consistent with the results at different time points, a significant increase in the GRP78 protein level was observed when the MOI was switched to 1.5, compared to the mock control (**Figure 5D**). Furthermore, RT-qPCR analysis showed that GRP78 mRNA expression levels were significantly elevated upon NDV infection at 24 hpi (**Figures 5E, F**). These results demonstrate that NDV infection leads to the upregulation of GRP78 at both protein and mRNA levels in HeLa and DF-1 cells infected with the virus.

### 3.5 siRNA-mediated knockdown of GRP78 suppresses NDV replication

**Figure 6:**
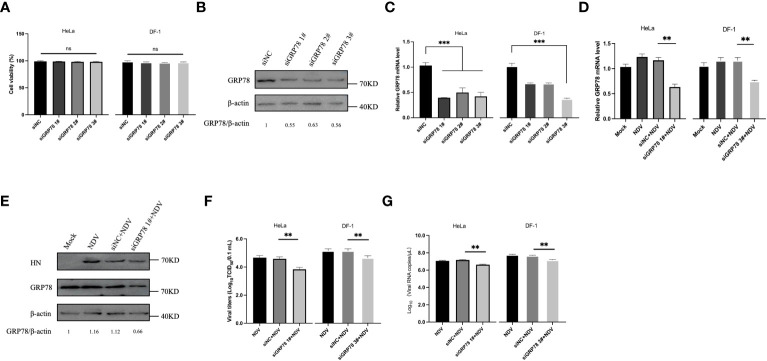
Knockdown of GRP78 inhibits NDV infection and replication. **(A)** Cytotoxicity assay of cells transfected with siRNA targeting GRP78. HeLa and DF-1 cells were transfected with siRNAs targeting GRP78 and siRNA-NC for 36 h, and the cell viability was assessed using the CCK-8 kit. **(B)** HeLa cells were transfected with GRP78 siRNAs for 36 h. GRP78 protein expression was analyzed by Western blotting. **(C)** HeLa cells and DF-1 cells were transfected with GRP78 siRNAs for 36 h. GRP78 mRNA levels were analyzed by RT-qPCR. **(D)** HeLa cells were transfected with either siRNA GRP78 1# or siNC, and DF-1 cells were transfected with either siRNA GRP78 3# or siNC for 36 h, followed by infection with NDV (MOI=1). The expression of GRP78 mRNA was quantitated by real-time PCR. **(E)** HeLa cells were transfected with either siRNA GRP78 1# or siNC for 36 h, and infected with NDV (MOI=1). The protein expression of GRP78 was analyzed by Western blotting. **(F)** The virus titers in the culture supernatant were determined by TCID_50_ assay. **(G)** The copy number of NDV was analyzed by real-time PCR. All results are presented as the mean ± SD of data from three independent experiments. **p < 0.01; ***p < 0.001; ns, p > 0.05.

The role of GRP78 in NDV infection and replication was then investigated by knockdown of GRP78 with siRNA. Cells transfected with specific siRNAs targeting GRP78 (siRNA GRP78 1#, siRNA GRP78 2#, or siRNA GRP78 3#) or a control nontargeting siRNA were harvested at 36 hours post-transfection, and protein and mRNA expression levels were analyzed by Western blotting and RT-qPCR, respectively. The effect of siRNA transfection on cell viability was first assessed using a CCK-8 experiment, demonstrating that that transfection with siRNA GRP78 did not significantly impact the cell viability of HeLa and DF-1 cells, compared to the siRNA control group (**Figure 6A**). Western blot analyses confirmed that the three specific siRNAs targeting GRP78 efficiently reduced the cellular GRP78 protein levels, while the nonspecific siRNA control did not (**Figure 6B**). Consistently, siRNA treatment resulted in a decrease in GRP78 mRNA levels in HeLa cells and DF-1 cells, particularly evident in cells transfected with siRNA human-GRP78 1# and siRNA chicken-GRP78 3# with the most significant reduction (**Figure 6C**). Next, the protein and mRNA expression levels of GRP78 were examined after NDV infection in control and GRP78 siRNA-treated HeLa cells, showing a noticeable decrease in total GRP78 mRNA levels in NDV-infected HeLa and DF-1 cells transfected with siRNA GRP78 1#, compared to control cells (**Figure 6D**). Additionally, Western blotting analysis revealed the reduced GRP78 protein levels in HeLa cells transfected with siRNA GRP78 1# compared to the siRNA control (**Figure 6E**).

To further investigate the impact of GRP78 knockdown on NDV replication, DF-1 cells were similarly treated with siRNA and infected with NDV, showing that knockdown of GRP78 specifically reduced NDV replication (**Figures 6F, G**). Collectively, these findings confirm that GRP78 knockdown has a negative impact on NDV replication, suggesting that GRP78 may play a positive regulatory role in the NDV infection.

### 3.6 Overexpression of GRP78 promotes NDV replication

**Figure 7:**
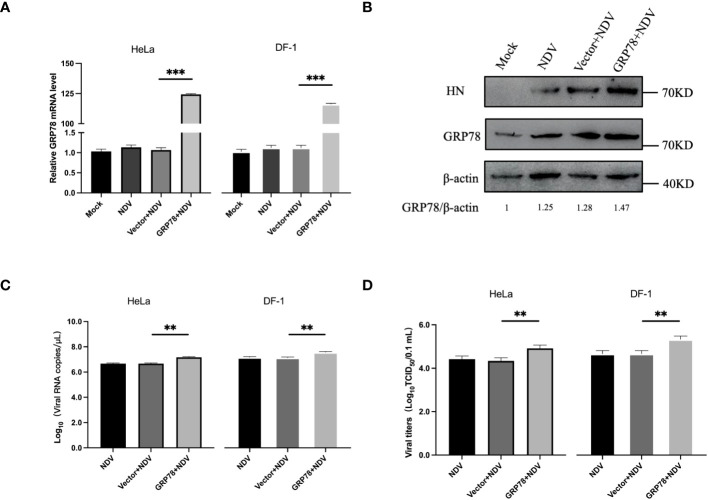
Overexpression of GRP78 promotes NDV replication. **(A)** HeLa and DF-1 cells were transfected with pXJ40-Flag-C-GRP78 or pXJ40 vector for 24 h and infected with NDV (MOI=1) for 24 h. The mRNA levels of GRP78 were analyzed by RT-qPCR. **(B)** The protein levels of GRP78 were detected by Western blotting in HeLa cells. **(C)** The copy number of NDV was analyzed by real-time PCR. **(D)** The virus titers in the culture supernatant were determined by TCID50 assay. All results are presented as the mean ± SD of data from three independent experiments. **p < 0.01; ***p < 0.001.

The effect of GRP78-HN interaction on NDV replication was then investigated by overexpression of GRP78 in HeLa and DF-1 cells. Cells were transfected with plasmids overexpressing GRP78 (pXJ40-Flag-C-GRP78) or empty vector control (pXJ40-Flag), respectively, infected with NDV at an MOI=1, and the protein and mRNA expression levels were analyzed by Western blotting and RT-qPCR. The results showed an increase in GRP78 mRNA levels in the infected cells, compared to the uninfected control cells (**Figure 7A**). As expected, HeLa cells overexpressing GRP78 exhibited significantly higher NDV replication, compared to cells transfected with the empty vector control (**Figure 7B**). Additionally, the viral titers were significantly higher in cells overexpressing GRP78, compared to the control cells (**Figures 7C, D**). These findings indicate that overexpression of GRP78 effectively promotes NDV replication in HeLa and DF-1cells, probably by facilitating NDV entry and enhancing viral replication.

### 3.7 GRP78 is required for effective viral attachment during NDV infection

**Figure 8:**
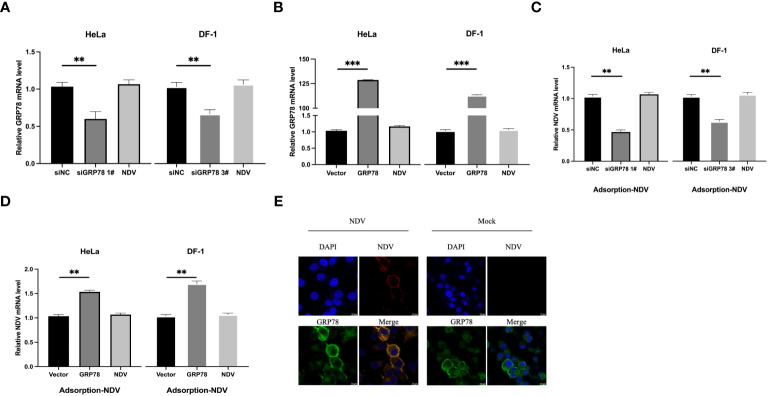
Functional involvement of GRP78 in the attachment of NDV to cells. **(A, B)** Effects of GRP78 knockdown and overexpression on the attachment of NDV to cells. HeLa and DF-1 cells were transfected with either siGRP78 for 36 h **(A)** or pXJ40-Flag-C-GRP78 for 24 h **(B)**, incubated with NDV (MOI=1) at 4°C for 1 h, and the GRP78 mRNA levels were determined by RT-qPCR. **(C, D)** HeLa and DF-1 cells were transfected with either siGRP78 for 36 h (C) or pXJ40-Flag-C-GRP78 for 24 h **(D)**, incubated with NDV (MOI=1) at 4°C for 1 h, and the NDV mRNA levels were determined by RT-qPCR. All results are presented as the mean ± SD of data from three independent experiments. **p < 0.01; ***p < 0.001. **(E)** Co-localization of GRP78 and NDV in HeLa cells during the attachment stage. HeLa cells were incubated with NDV (MOI=10) at 4°C for 1 h, followed by immunostaining with rabbit anti-GRP78 pAbs (red) and rabbit anti-NDV pAb (green), respectively, and examined by confocal microscopy. Nuclei were stained with DAPI (blue).

To pinpoint the functional involvement of GRP78-HN interaction in the exact step(s) of the NDV replication cycle, we first examined the effects of overexpression and knockdown of GRP78 on the viral attachment to cells. HeLa cells were transfected either with siGRP78s for 36 h or pXJ40-Flag-C-GRP78 for 24 h, and incubated with NDV at an MOI=1 at 4°C for 1 h to allow viral attachment. The mRNA levels of GRP78 and NDV were measured using RT-qPCR, showing corresponding decreased or increased levels of GRP78 mRNA in the knockdown or overexpression cells (**Figures 8A, B**). Similarly, the mRNA levels of NDV exhibited the same pattern, demonstrating that knockdown of GRP78 reduced the attachment of NDV, and overexpression of GRP78 enhanced the adsorption of NDV in HeLa cells (**Figures 8C, D**). These findings suggest that GRP78 is involved in the process of NDV attachment.

To examine whether GRP78 co-localized with NDV virions during the adsorption step, HeLa cells were inoculated with NDV at 4°C for 1 h to allow viral attachment, and their co-localization was examined via confocal microscopy. The results demonstrated the co-localization of GRP78 and HN on the cell surface, supporting that the direct interaction between GRP78 and HN would enhance NDV infection by increasing viral attachment (**Figure 8E**).

**Figure 9:**
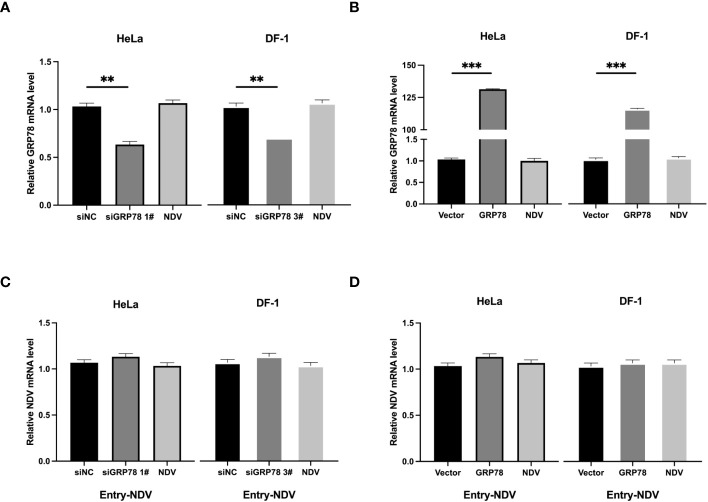
Effects of GRP78-knockdown and overexpression on the entry of NDV. **(A, B)** HeLa cells and DF-1 cells were transfected with either siGRP78 for 36 h **(A)** or pXJ40-Flag-C-GRP78 for 24 h **(B)**, incubated with NDV (MOI=1) at 4°C for 1 h, followed by shifting to 37°C for another 1 h after thorough washing. GRP78 mRNA levels were determined by RT-qPCR. **(C, D)** HeLa cells and DF-1 cells were transfected with either siGRP78 for 36 h **(C)** or pXJ40-Flag-C-GRP78 for 24 h **(D)**, incubated with NDV (MOI=1) at 4°C for 1 h, followed by shifting to 37°C for another 1 h after thorough washing. NDV mRNA levels were determined by RT-qPCR.All results are presented as the mean ± SD of data from three independent experiments. **p < 0.01; ***p < 0.001.

To further assess whether GRP78 facilitates viral entry, HeLa and DF-1 cells were similarly transfected and incubated with NDV at 4°C for 1 h, and then shifted to 37°C for another 1h after thorough washing to allow viral entry. After the cells were washed once with a citrate buffer and three times with PBS, the level of virus entry was evaluated by measuring the mRNA levels of GRP78 and NDV. Consistent with the previous results, knockdown or overexpression of GRP78 significantly altered its mRNA levels (**Figures 9A, B**), however, GRP78 knockdown or overexpression did not significantly affect the NDV mRNA levels (**Figures 9C, D**). These findings indicate that GRP78 does not promote the entry of viral RNA, but rather plays a role in the viral adsorption step.

### 3.8 Incubation of cells with GRP78 polyclonal antibodies inhibits NDV infection

**Figure 10:**
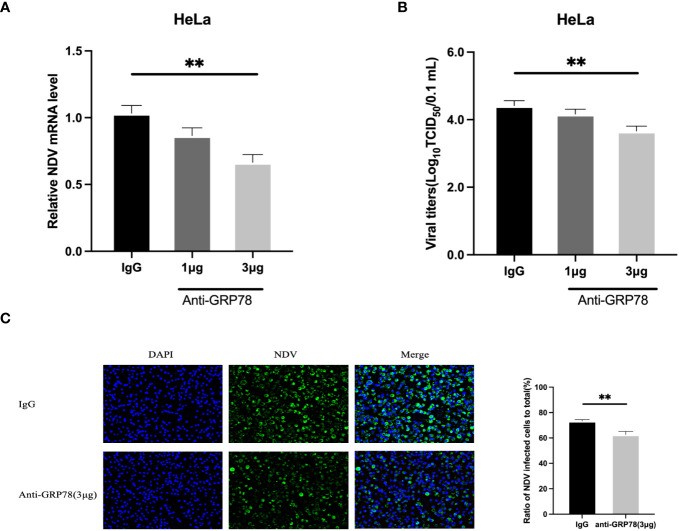
GRP78 antibody inhibits NDV infection in HeLa cells. **(A, B)** Inhibition of NDV infection by GRP78 pAbs. HeLa cells were incubated with varying concentrations of GRP78 pAbs or rabbit IgG for 1 h at 37°C, and incubated with NDV (MOI=1) for 1 h at 4°C. After 24 hpi, cells and supernatants were harvested separately, and the levels of NDV mRNA were determined using RT-qPCR **(A)**, and the virus titers in the supernatants were determined using TCID50 assay **(B)**. All results are presented as the mean ± SD of data from three independent experiments. **, p < 0.01. **(C)** NDV infectivity was determined by immunofluorescence assay (IFA) using rabbit anti-HN pAbs (green). Nuclei were stained with DAPI (blue).

To investigate if masking of GRP78 expressed on the cell surface by incubation with a GRP78 antibody would affect the NDV infection, HeLa cells were pre-treated with 1 and 3μg of rabbit anti-GRP78 pAbs or rabbit IgG antibodies (control) at 37°C for 1 h, followed by incubation with NDV at 4°C for 1 h. After washing and incubation in DMEM at 37°C for 24 h, cells and culture supernatants were separately collected for analysis of NDV mRNA levels by RT-qPCR and virus titer by TCID_50_ assay. The results revealed that pre-incubation of HeLa cells with rabbit anti-GRP78 pAbs resulted in the detection of significantly lower levels of viral mRNA, compared to cells pre-incubated with rabbit IgG (**Figure 10A**). Similarly, pre-incubation of HeLa cells with rabbit anti-GRP78 pAbs significantly reduced the virus titer and infectivity compared to the control (**Figures 10B, C**). These findings confirm that treatment of HeLa cells with GRP78 antibodies inhibited NDV infection and replication.

## 4 Discussion

NDV is a highly contagious virus that infects domestic and wild birds globally, causing a wide range of clinical symptoms from mild respiratory to severe neurological manifestations and mortality. Elucidation of a fuller interactome between NDV proteins and host cell surface molecules would be essential for a deeper understanding of the viral pathogenesis, host range and tissue and cell tropisms, and was a focus of the NDV research in the past three decades (30–32). In this study, we report the identification of 167 host proteins that may interact with the HN protein. Through bioinformatics analysis, we elucidated the biological functions of these interacting proteins, revealed their involvement in NDV-related biological processes, and constructed an HN-host interaction network. Further detailed analysis of the interaction between GRP78 and HN confirmed the direct interaction of the two proteins and mapped the domain responsible for this binding to the N-terminal 1-326 amino acid region in GRP78. NDV infection of HeLa and DF-1 cells was shown to upregulate and induce the expression of GRP78 on the cell surface, where it co-localized with HN and mediated NDV adsorption. This conclusion was further supported by the observation that incubation of cells with specific antibodies would mask GRP78 expressed on the cell surface and reduce the viral attachment, leading to the suppression of NDV infection. GRP78 may, therefore, cooperate with other receptors and cell surface molecules during the early stage of NDV infection to promote viral replication and infection.

Multiple cell surface receptors, including proteins modified by sialic acid and other glycosaminoglycans, have been identified as key factors for binding of specific viral proteins to cell surface and for mediating viral entry into host cells (33–35). HN, the transmembrane glycoprotein, is critical in the initial attachment of NDV to host cells. Given its significance in NDV pathogenesis and its potential as a target for antiviral therapies, extensive research has been conducted on this protein. It binds to receptors on the cell surface that contain sialic acid, facilitating the viral entry into host cells (36, 37). Additionally, HN possesses a neuraminidase activity that aids the release of newly formed viral particles from infected cells by cleaving the sialic acid residues from host cell surface receptors, promoting the release of viral particles and the spread of infection to neighboring cells (38–40). This protein shows considerable variability among different strains and plays a crucial role in determining the viral host range and tissue tropism. On the other hand, direct interaction of some cell surface molecules, such as 1-formyl-β-carboline derivatives, with HN was shown to negatively influence the adsorption of NDV (41). One specific example was CG-1B with an anti-NDV activity via binding to the N-glycans present on the HN glycoprotein (42).

As binding of HN to host cell receptors is a main step that initiates the infection process, the host range and tissue tropism of NDV are mainly determined by the specific interactions between this viral transmembrane glycoprotein and host cell receptors as well as other surface molecules (43, 44). In addition to gaining a deeper understanding of the molecular mechanisms underlying the viral pathogenesis, NDV-based targeted therapies have been developed based on the specific interaction between HN and cellular receptors. For example, an antagonist of α7 nAChR holds promise as a molecular target for lung adenocarcinoma therapy (45). By regulating the α7 nAChR signaling pathways, a recombinant NDV, rL-RVG, was constructed and was shown to enhance apoptosis and inhibit the migration of A549 lung adenocarcinoma cells (45). Further elucidation of the specificity of NDV proteins-cellular receptor interactions would expand the potential of using NDV as a therapeutic agent.

The ER chaperone protein GRP78 has recently emerged as a significant player in viral infection. Multiple studies have demonstrated the dependence of various viruses, including Ebola virus, hepatitis C virus and SARS-CoV-2, on GRP78 for host cell entry (46–51). The interaction between GRP78 and viral surface proteins, such as the Ebola virus glycoprotein and the SARS-CoV-2 spike protein, facilitates viral attachment and subsequent entry into the host cell. Moreover, the upregulation of GRP78 in the response to viral infection may render infected cells a survival advantage by promoting cell survival and preventing apoptosis (52–55). GRP78 may also act as a restriction factor for viral replication by binding to viral proteins and impeding their proper folding or assembly (56–59). Modulating the expression and functions of GRP78 may also influence the immune response to viral infection. For example, treatment of NDV-infected cells with LiCl significantly reduced the transcript and protein levels of GRP78, providing cellular protection against the ER stress induced by NDV infection (60). The flux of HN through the ER in simian virus 5 (SV5)-infected cells can trigger the activation of GRP78-BiP transcription (61).

Targeting the replication cycle of NDV represents a promising approach for developing antiviral drugs, by focusing on viral proteins such as F and HN proteins (62, 63). By targeting these proteins, it may be possible to hinder NDV replication and mitigate the severity of the infection. Another avenue worth exploring is targeting specific cell surface proteins involved in the viral entry and infection. For instance, NDV relies on the binding to sialic acid-containing receptors on the cell surface to infect host cells (64). Developing drugs that interfere with the interaction between NDV and these receptors, or targeting other cell surface proteins involved in the viral entry, would serve as effective strategies for prevention and treatment of NDV infection. The identification of the N-terminal 326 amino acid region in GRP78 as a domain essential for its interaction with HN would open a potential avenue for the design of such an intervention.

## 5 Conclusions

This study reveals an important role played by the molecular chaperone GRP78/BiP during the initial stage of NDV infection, providing valuable insights into NDV-host interactions and contributing to our understanding of the pathogenesis and replication mechanisms of NDV.

## Data availability statement

The mass spectrometry proteomics data have been deposited to the ProteomeXchange Consortium (http://proteomecentral.proteomexchange.org) via the iProX partner repository with the dataset identifier PXD045966.

## Author contributions

CH: Conceptualization, Data curation, Investigation, Writing – original draft. ZX: Conceptualization, Writing – review & editing. YL: Methodology, Project administration, Writing – review & editing. DL: Funding acquisition, Supervision, Writing – review & editing. RC: Funding acquisition, Validation, Writing – review & editing.
